# Dkk1: A key molecule in joint remodelling and fibrosis

**DOI:** 10.31138/mjr.28.4.174

**Published:** 2017-12-22

**Authors:** Kalliopi Klavdianou, Stamatis-Nick Liossis, Dimitrios Daoussis

**Affiliations:** Department of Rheumatology, University of Patras Medical School, Patras University Hospital, Patras, Greece

**Keywords:** Dkk-1, fibrosis, inhibitor, joint remodeling

## Abstract

Dickkopf-1 (Dkk-1) is a Wnt signaling pathway inhibitor that has been shown to play an important role in joint remodeling, in experimental models of arthritis and in humans. Recent data suggest that this molecule is involved in the fibrotic process as well.

**OBJECTIVES:**

This review summarizes all the available data regarding the role of Dkk-1 in joint remodeling and fibrosis.

**METHODS:**

An electronic search in literature was performed using the terms Dickkopf-1 (Dkk-1), fibrosis, Systemic Sclerosis (Scleroderma), joint remodeling, Ankylosing Spondylitis. Moreover, references in the retrieved articles were reviewed.

**RESULTS:**

Dkk1 expression seems to determine the fate of an arthritic joint, shifting the process of joint remodelling towards either an erosive/destructive phenotype, when Dkk1 is overexpressed or new bone formation when its expression is decreased. In humans, evidence suggests that Dkk-1 may be dysfunctional in patients with ankylosing spondylitis. Moreover, data from animal models indicate that Dkk-1 may be crucially involved in the fibrotic process in several organs such as the liver, lungs and kidneys. In animal models, enhanced expression of Dkk-1 had a clear suppressive effect on fibrosis suggesting that this molecule could be an attractive target in fibrotic diseases. In humans, Dkk-1 is clearly expressed in the skin. However, in patients with systemic sclerosis Dkk-1 is strikingly absent from the skin.

**CONCLUSION:**

Dkk-1 plays a critical role in joint remodeling and fibrosis and could serve as either a biomarker in diseases characterized by pathological joint remodeling or as a potential therapeutic target in fibrotic diseases.

Recent data suggest that reactivation of developmental pathways is a common feature of both ectopic ossification and fibrosis. These pathways are mainly active during embryonic development and are necessary for homeostasis, development and healing.^1–4^ However, they can reactivate in adulthood as a response to tissue damage. The best studied developmental pathway is the so called canonical Wnt pathway, mediated by β-catenin.^5^ The Wnt pathway was firstly linked to osteoblastogenesis, when researchers found that gain or loss of function mutations of LRP5 (low-density lipoprotein related receptor 5-the main receptor mediating Wnt signaling), lead to high or low bone mass, respectively.^6,7^ On the other hand, the link between the Wnt pathway and fibrosis became apparent when a high number of mutations in β-catenin and its negative regulator APC (adenomatosis polyposis coli) in aggressive fibromatosis was detected.^8^ Dickkopf-1 (Dkk-1) is a soluble inhibitor of Wnt pathway. Data suggest that Dkk-1 regulates several aspects of bone biology^9^ and fibrosis.^10^ More specifically, it has been shown that Dkk-1 is a master regulator of joint remodeling in animal models of arthritis shifting the balance towards new bone formation when its expression is decreased and towards joint destruction when its expression is increased.^11^ It has been recently shown that Dkk-1 mediated Wnt inhibition could suppress TGF-β mediated fibrosis.^10^

In this review we summarize and discuss all available data regarding the role of Dkk-1 in joint remodeling and fibrosis **(Figure 1)**. These two processes are of importance in the field of rheumatology. Spondyloarthropathies (SpA) are characterized by ectopic ossification/new bone formation. Systemic sclerosis (SSc) is the prototypic systemic fibrotic disease. Ectopic ossification (a hallmark of SpA) and fibrosis (a hallmark of SSc) are both tightly linked to the Wnt pathway.

**Figure 1:**
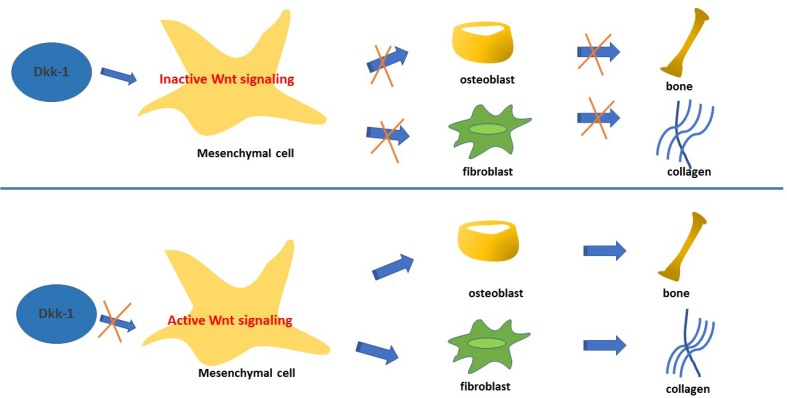
Dkk-1 and mesenchymal cell differentiation. Binding of Dkk-1 to its receptors on mesenchymal cell inhibits Wnt signaling and differentiation of the cell into either fibroblast or osteoblast (upper panel). Dkk-1 does not bind to its receptors. Wnt ligands bind to their receptors on mesenchymal cell. Wnt signaling is active and controls the differentiation of mesenchymal cell into either fibroblast or osteoblast (lower panel).

## METHODS

An electronic search in literature was performed using the terms Dickkopf-1 (Dkk-1), fibrosis, Systemic Sclerosis (Scleroderma), joint remodeling, Ankylosing Spondylitis. Moreover, references in the retrieved articles were reviewed.

Dkk-1 measured by sandwich ELISA is referred to as “circulating”, while Dkk-1 measured by functional ELISA, with human LRP6 coated plates, measuring the ability of Dkk-1 to bind Lrp6 receptor, is referred to as “functional”.

### The canonical Wnt signaling pathway-Short overview

**Figure 2:**
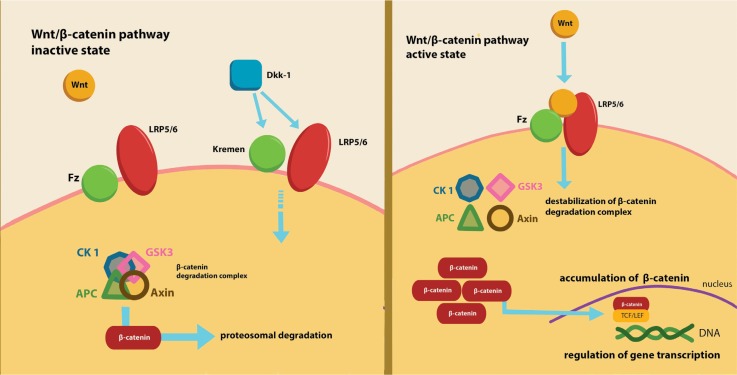
The canonical Wnt/β-catenin signaling In the absence of Wnt ligand, β-catenin protein is constantly degraded by the action of a complex, composed of casein kinase 1 (CK1), glycogen synthase kinase 3 (GSK3), the scaffolding protein Axin and adenomatous polyposis coli (APC). Dkk-1 interacts with Lrp5/6 and the transmembrane protein Kremen. Dkk-1, LRP5/6 and Kremen form a complex that disrupts Wnt/LRP6 signaling by promoting endocytosis and removal of the Wnt receptor from the plasma membrane (left panel). When a ligand is present, it binds to its coreceptor complex consisting of LRP5/6 and Frizzled (Fz). Degradation complex is destabilized and β-catenin degradation is blocked. This leads to accumulation of β-catenin in cytoplasm; the active β-catenin translocates to the nucleus, where it acts as a transcriptional coactivator with TCF/LEF to activate Wnt target genes (right panel).

Wnt proteins are the ligands of the pathway. Nineteen Wnt genes have been identified. These secreted factors bind to a family of Frizzled transmembrane receptors, which format a complex with the LRP5/6 coreceptor. In absence of a Wnt ligand, β-catenin cytoplasmic resources are constantly depleted via a protein complex phosphorylating β-catenin, leading to proteasomal degradation. When a Wnt ligand is present the proteins of this ‘destruction’ complex are directed towards the membrane, allowing β-catenin to accumulate in the cytoplasm and move to the nucleus. In the nucleus, β-catenin cannot bind directly to DNA but it binds to the complex of transcription factors TCF-LEF (Lymphocyte Elongation Factor) driving the transcription of several target genes **(Figure 2)**. The pathway is also extracellularly regulated by several inhibitors. Dkk-1 is an inhibitor of the pathway; the most cited view on how this inhibition is performed is that Dkk-1 forms a trimer complex with the membrane protein Kremen and LRP6, promoting endocytosis and depletion of cell surface from LRP5/6 receptors; the latter are not available for binding the Wnt proteins.^12^

### Dkk-1 in animal models of inflammatory arthritis

Diarra and co-workers identified Dkk-1 as a key molecule for joint remodeling in arthritis.^11^ They used an anti-Dkk-1 mAb in several animal models of inflammatory arthritis and found that Dkk-1 blockade led not only to inhibition of bone erosions in these mice, but to osteophyte formation, as well. Their study provided experimental evidence suggesting that Dkk-1 can shift the balance towards either new bone formation when its expression is low or erosion/joint destruction when the molecule is overex-pressed.

Walsh and coworkers tried to explore why repair of erosions is inadequate in Rheumatoid Arthritis (RA)^13^. Using dynamic bone histomorphometry in a murine model of arthritis, they found that bone formation rates at bone surfaces adjacent to inflammation were similar to those observed in nonarthritic bone, indicating that osteoblast activity does not compensate for bone loss at these sites. qRT-PCR showed that DKK1 mRNA expression was elevated in arthritic synovial tissues above the expression levels in nonarthritic mice, with maximum expression when inflammation and focal bone erosion were at peak. These data indicate that focal bone loss in RA is not only due to osteoclast activation but suppression of osteoblastogenesis also contributes. The role of Dkk-1 in sacroiliitis, was explored by Uderhardt et al.^14^ For this purpose they used TNFtg mice, which develop inflammation and erosions at the sacroiliac joints. Administration of anti-Dkk-1 mAb to these mice led to attenuation of erosions and fusion of the sacroiliac joints. B-catenin expression assessed by immunohistochemistry, was upregulated within the sacroiliac joints upon Dkk-1 blockade, suggesting that Dkk-1 inhibition leads to enhancement of Wnt signaling and consequently to new bone formation.

### Dkk-1 in Ankylosing Spondylitis

Diarra was the first to assess functional Dkk-1 levels in patients with Ankylosing Spondylitis (AS).^11^ AS patients had lower functional Dkk-1 levels than patients with RA and healthy subjects, suggesting a link between joint remodeling and Dkk-1 serum levels. Our research group has found that circulating Dkk-1 levels are higher in patients with AS than patients with RA and healthy controls.^15^ We performed a functional ELISA and confirmed that functional Dkk-1 levels are decreased in AS. We next tried to investigate the reason of the discordance between functional and circulating levels of Dkk-1 in AS. For this purpose, Jurkat T cells were cultured and treated with serum from either AS patients or healthy subjects. The addition of AS serum in the culture resulted in higher Wnt pathway activation, compared with addition of healthy subjects’ serum. Addition of a mAb that specifically blocks the interaction of Dkk-1 with its receptor in control serum–treated cells, led to a significant increase in Wnt signaling, as indicated by active β-catenin levels. In contrast, addition of the neutralizing anti-human Dkk-1 mAb to AS serum–treated Jurkat cells did not affect Wnt pathway activation, confirming our hypothesis that Dkk-1 is dysfunctional in Ankylosing Spondylitis. Several other small studies have investigated the association between Dkk-1 serum levels and AS with various results.^16,17^ However, a recent meta-analysis of seven case-control trials with a total of 300 AS patients, 136 RA patients and 232 healthy controls has confirmed that circulating Dkk-1 levels were significantly higher in AS patients than in normal controls.^18^

A large scale French study assessed circulating levels of Dkk-1 in a cohort of 486 patients (DESIR cohort) with early axial SpA and found that circulating Dkk-1 levels were increased in these patients compared with healthy subjects and positively correlated with female gender, CRP levels and sacroiliitis. The authors found no association between DKK-1 SNPs (single nucleotide polymorphisms), Dkk-1 circulating levels and structural damage indicating that in AS Dkk-1 circulating levels are not determined genetically.^19^

Heiland et al has previously described that functional Dkk-1 levels had a protective role against radiological progression in patients with AS, whereas circulating Dkk-1 was not predictive.^20^ A recent study confirmed these findings^21^ indicating that Dkk-1 is candidate biological marker for structural progression in AS.

### Dkk-1 in Rheumatoid Arthritis

Diarra et al. found that patients with RA had higher functional Dkk-1 levels than patients with AS and healthy subjects.^11^ Since then, several studies assessed circulating Dkk-1 levels in patients with RA in an effort to explore whether this molecule can serve as a prognostic marker for joint destruction. Most studies reported higher Dkk-1 circulating levels compared to healthy subjects; increased levels correlated with high rates of erosions and joint destruction^22–24^ indicating that Dkk-1 may be a useful biomarker for radiological progression in RA Synovial fibroblasts play an important role in joint destruction and regulation of inflammatory infiltration in RA.^25^ However, disease mechanisms during the early stages of RA remain unclear. Recently, researchers studied the potential role of Dkk-1 derived from synovial fibroblasts in patients with very early RA.^26^ Fibroblasts were isolated from treatment naïve patients with RA, with symptoms of ≤12 weeks’ duration. At 18-month follow-up these patients had either resolving arthritis or RA. Synovial fibroblasts of patients with a diagnosis of RA expressed significantly higher DKK1 mRNA levels at baseline than patients finally diagnosed with resolving arthritis, suggesting that even at this very early stage, synovial fibroblasts may have gained the capacity to impair bone repair and induce bone erosion through increased expression of Dkk-1.

Increased expression of Dkk-1 in RA could partially be genetically controlled as suggested by a study by de Rooy et al.^27^ In this study, 3 DKK1 SNPs were associated with structural progression. However, these results were not replicated in a large Spanish prospective cohort.^24^

### The effect of proinflammatory cytokines on Dkk-1 expression

Dkk-1 production has been shown to be up-regulated by TNFα.^11^ More specifically, Diarra et al suggest that bone formation is hampered by the TNFα-mediated expression of Dkk-1, which suppresses Wnt signaling. Blockade of Dkk-1 relieves Wnt signaling from Dkk-1-mediated suppression and induces bone formation.

Our group has previously shown that TNFα inhibitors have a different effect on circulating Dkk-1 levels in patients with RA and AS.^15^ Circulating Dkk-1 levels increase in patients with AS and decrease in RA patients following anti-TNFα treatment. Since TNFα upregulates Dkk-1, post- treatment Dkk-1 increase is unexpected in AS. One could speculate that this increase is a counterbalancing protective mechanism to downregulate Wnt signaling and inhibit new bone formation.

In an effort to explore the strong link between Dkk-1 expression and the joint remodeling phenotype, Yeremenko and coworkers analyzed the expression of Dkk-1 and its regulation by proinflammatory cytokines in the inflamed peripheral joints of patients with SpA and RA.^28^ Expression of Dkk-1 and proinflammatory cytokines were assessed in synovial fluid (SF) and synovial tissue while regulation of Dkk-1 production by proinflammatory cytokines was assessed in fibroblast-like synoviocyte (FLS) cultures. Dkk-1 circulating levels and DKK1 mRNA levels showed an inverse correlation with IL-6 levels in both SpA SF and RA SF. They next investigated whether TNFα and IL-6 regulate Dkk-1 production by FLS *in vitro*. TNFα stimulation induced a 2-fold increase in Dkk-1 production by FLS. In contrast, IL-6 significantly suppressed Dkk-1 production by FLS, suggesting that these cytokines have opposite effects on Dkk-1 expression and their balance could determine the fate of an inflamed joint.

### Dkk-1 in fibrosis

The mechanisms causing aberrant fibroblast activation and extracellular matrix deposition in fibrosis are incompletely understood. Accumulating evidence suggests though that Wnt signaling has an important role in fibro-genesis. Several members of the Wnt pathway appear to be upregulated in animal models of sclerodema^4,29^ and in fibroblasts from patients with SSc.^30^ Moreover, lung fibroblasts have been shown to respond to Wnt pathway stimulation.^31,32^ In renal cells, the Wnt pathway promotes the expression of numerous fibrosis-related genes, while Wnt signaling inhibition ameliorates kidney injury and attenuates renal fibrotic lesions in several models of chronic kidney disease.^33^ Wnt signaling has also been implicated in the pathogenesis of liver fibrosis^34^ and in scarring after myocardial infarction.^35^ Therapeutic interventions targeting Wnt signaling in fibrotic diseases may achieve regression of fibrosis. Along this line of thought researchers tried to identify the role of Dkk-1 in fibrotic diseases.

#### Pulmonary fibrosis

Abberant activation of the Wnt pathway has been associated with pulmonary fibrosis. Several studies have linked increased Wnt/β-catenin signaling to the pathogenesis of idiopathic pulmonary fibrosis (IPF).^36–38^ Pfaff and coworkers analyzed the expression, localization and function of Dkk-1 in IPF lungs.^39^ DKK1 had significantly increased mRNA and protein expression in fibrotic compared to healthy lungs. Immunohistochemistry revealed that Dkk-1 was predominantly localized in basal bronchial epithelial cells. Moreover, Dkk-1 expression assessed by ELISA, presented a 2-fold increase in the bronchoalveolar lavage fluid (BALF) of patients with IPF patients compared to patients with chronic bronchitis and healthy subjects. Finally, they performed functional studies using human bronchial and alveolar epithelial cell lines. They found that WNT-induced epithelial cell proliferation was attenuated by the addition of Dkk-1. These findings indicate that Dkk-1 may modulate bronchial epithelial cell maintenance.

It is known that acute lung injury may lead to fibrogenesis. Sun and coworkers conducted a study to determine the effects of bone marrow-derived mesenchymal stem cells (MSCs) in a model of HCl-induced acute lung injury in rats.^40^ They found that Wnt signaling may affect the differentiation of MSCs *in vivo* and *in vitro*; activation of Wnt signaling as evaluated by β-catenin expression in whole cell lysates, attenuated the epithelial differentiation of MSCs cocultured with epithelial cells. By contrast, Dkk-1 mediated inhibition of Wnt/β-catenin signaling prevented myofibroblast differentiation of MSCs, suggesting that inhibiting of Wnt/β-catenin signaling via Dkk-1 may be a therapeutic approach for lung injury or pulmonary fibrosis.

#### Renal fibrosis

The role of Wnt signaling in kidney fibrosis has been established;^41^ however, the role of Dkk-1 in this process has not been extensively explored. Wnt/β-catenin signaling promotes renal interstitial fibrosis in mice following renal injury.^42,43^

In a study by He et al., all members of the Wnt family were upregulated in the kidneys of mice following obstructive injury and Wnt signaling was increased in renal tubular epithelial cells. Intravenous injection of naked plasmid vector encoding DKK1 gene inhibited Wnt signaling as assessed by Western blot analysis and immunohistochemistry. This led to a significant suppression of renal α-SMA, type I collagen and fibronectin mRNA expression, in obstructed kidney, indicating that DKK1 gene therapy inhibits myofibroblast activation.^42^ Another study showed that Dkk-1 inhibited pericyte activation and transition to myofibroblasts in murine kidneys.^44^

Wnt-β-catenin signaling pathway has also been demonstrated to be involved in endothelial to mesenchymal transition, a key event in fibrotic diseases such as diabetic renal fibrosis.^45^

#### Liver Fibrosis

Liver fibrosis is a common wound-healing response to chronic liver injury. Activation of hepatic stellate cells (HSC) and differentiation to myofibroblasts is the key event in liver fibrogenesis. Wnt signaling promotes hepatic fibrosis by enhancing HSC activation and survival.^46^ However, the role of Dkk-1 in liver fibrosis has not been extensively explored.

Cheng et al investigated the expression of Wnts, Frizzled receptors and coreceptors in culture-activated HSCs obtained from either cholestatic or control rats.^47^ mRNA levels of Wnt ligands and coreceptors were increased in activated HSCs compared to quiescent HSCs. Wnt signaling was induced by Wnt1 and inhibited by Dkk-1. In activated HSCs, DKK1 adenovirus infection could enhance the promoter activity of the PPARγ-driven PPAR response element (PPRE), which is a key adipogenic transcriptional regulator, and restore HSC quiescent state. Dkk-1 mediated restoration of PPAR-γ activity in activated HSCs possibly takes place through epigenetic modifications.^48^

#### Pancreatic fibrosis

The persistent activation of pancreatic stellate cells (PSCs) plays a crucial role in pancreatic fibrogenesis.^49^ Hu et al explored the role of Wnt signaling during PSC activation in chronic pancreatitis(50). Pancreatitis was elicited in mice by intraperitoneal injections of caerulein. They confirmed that Wnt/β-catenin pathway is activated during the activation of mouse PSCs *in vitro.* The addition of recombinant mouse Dkk-1 in cell cultures led to Wnt signaling downregulation, inhibited the activation of PSCs and reduced collagen synthesis. The authors suggest that an imbalance in Wnt/Dkk-1 negative feedback signaling promotes the persistent activation of PSCs and pancreatic fibrosis.

#### Dkk-1 in Systemic sclerosis

Systemic sclerosis (SSc) is the prototypical systemic fibrotic disease. Even though its pathogenesis remains largely unknown a lot of evidence points to the direction of TGF-β as a key molecule.^51^ Akhmetshina et al identified a novel link connecting TGF-β and the canonical Wnt pathway.^10^ Firstly, they confirmed that Wnt signaling is active in fibrosis by assessing the nuclear accumulation of β-catenin in human fibrotic tissue of patients with SSc and in fibroblasts of different experimental mouse models of dermal, liver and pulmonary fibrosis. They next investigated whether inhibition of the canonical Wnt pathway blocks the development of fibrosis. Dkk-1 transgenic (Dkk-1 tg) mice were resistant to bleomycin-induced fibrosis and Dkk-1 prevented fibrosis in Tsk-1 mice. The authors suggest that this protective effect of Dkk-1 on experimental fibrosis is linked to TGF-β. They present experimental data that TGF- β activates Wnt signaling through inhibition of Dkk-1 expression. Epigenetic alterations are possibly involved in the downregulation of Dkk-1 expression in fibrosis. In a recent study, the promoters of DKK1 and SFRP1 were found to be hypermethylated in fibroblasts and peripheral blood mononuclear cells of patients with SSc(52). Promoter hypermethylation resulted in impaired transcription and decreased expression of DKK1 and SFRP1 in SSc. Inhibition of DNA methyltransferases in fibroblasts obtained from either patients with SSc or mice with bleomycin-induced fibrosis led to increase in the expression of DKK1 and SFRP1 and effectively ameliorated experimental fibrosis, by inhibiting canonical Wnt signaling.

Experimental^53–58^ and clinical^59–67^ data suggests that B cell depletion therapy may have a beneficial effect on skin and pulmonary fibrosis in patients with SSc. Our group investigated the hypothesis that Rituximab (RTX) exerts its antifibrotic effects through inhibition of the Wnt/β-catenin signaling.^68^ We found that Dkk-1 was clearly expressed in skin biopsies obtained from healthy subjects; however, it was absent in all baseline biopsies obtained from patients with SSc. However, following RTX treatment, half of the patients exhibited Dkk-1 expression in the follow up skin biopsies; upregulation of Dkk-1 expression highly associated with enhanced resolution of skin fibrosis. In contrast, Dkk-1 was not expressed in the follow up skin biopsies in scleroderma patients who received conventional therapy. Dkk-1 gene expression was 4.1-fold downregulated in scleroderma compared to healthy fibroblasts but was 4.2-fold upregulated, following RTX treatment. Taking into account the data indicating that TGFβ regulates Dkk-1 expression,^10^ we explored whether TGFβ skin expression is modified following B cell depletion therapy. TGF-β expression in fibroblasts from SSc patients was significantly decreased following RTX treatment and this downregulation was more pronounced in the subset of SSc patients with upregulation of Dkk-1. In our study we provide evidence, at both protein and gene expression level, that RTX treatment may affect Dkk-1 skin expression in patients with SSc and this may be a TGFβ-dependent effect.

## DISCUSSION

The role of Dkk-1 in joint remodeling has been well established, especially in animal models. Dkk-1 has been identified as a potential regulator of the balance between joint destruction and new bone formation in inflammatory arthritis.^11^ Human data are in accordance with the data from animal models. In RA, a disease characterized by erosions, functional Dkk-1 levels are elevated. In AS, a prototype bone forming disease, functional Dkk-1 levels are decreased and the molecule seems to be dysfunctional. The critical role of Dkk-1 in the joint remodeling process has made it an attractive therapeutic target. However, one should take into consideration that Dkk-1 is a Wnt pathway inhibitor, and Wnt pathway controls several aspects of human homeostasis. So far, data from animal models are encouraging regarding the safety profile of such targeting.^69^

Several therapeutic agents that specifically target the Wnt pathway are under development;^70^ however it is still early to know whether they bear an acceptable safety profile. Romosozumab, a monoclonal antibody that binds sclerostin, an inhibitor of the Wnt pathway is already tested in phase 3 studies for osteoporosis therapy, showing a good safety profile.^71^ The fact that BHQ880, an antiDkk-1 mAb, was well-tolerated in a phase Ib study in patients with relapsed or refractory multiple myeloma and prior skeletal-related events is encouraging for the safety of Dkk-1 inhibition.^72^ A phase I study evaluating safety and tolerability of an anti-Dkk-1 mAb(RN564) in women with osteopenia and healthy men has been completed and the results are expected.^73^

The next question is whether Dkk-1 could serve as biomarker for radiographic progression in either AS or RA. Serum functional^21^ and circulating^74^ Dickkopf-1 levels tend to be inversely correlated with radiographic severity of ankylosing spondylitis. Moreover, data suggest that patients with RA and increased Dkk-1 levels present rapid radiographic progression.^22^ Large-scale studies are needed before this molecule can be accepted as a validated biomarker in clinical practice.

Several studies have highlighted a key role for canonical Wnt signaling in fibrosis of several organs such as the heart, lungs, kidneys and skin (**Table 1**). Since aberrant activation of Wnt signaling can be a result of silencing of endogenous Wnt antagonists, such as Dkk-1, investigators have explored the role of this molecule in fibrosis. Data from animal models suggest that Dkk-1 is highly effective in antagonizing Wnt signaling in fibrosis. The recent data that TGF-β inhibits Dkk-1 resulting in abberant Wnt activation and fibrosis^10^ suggests that inhibition of the canonical Wnt pathway via Dkk-1 might be an effective approach to target TGF-β signaling in fibrotic diseases.

**Table 1.** The major clinical entities in which Wnt signaling is involved and Dkk-1 levels.

**Disease**	**Wnt signaling involvement**	**Dkk-1 levels**
Ankylosing Spondylitis	unclear involvement	↓functional^11,15,18,19^ ↑ circulating
Rheumatoid Arthritis	unclear involvement	↑functional^11^ ↑ circulating^22–24^
Systemic Sclerosis	↑^10,30^	↓DKK-1 gene expression in fibroblasts Absent from skin^52^
Pulmonary fibrosis	↑^36–40^	↑DKK-1mRNA and protein expression in fibrotic lungs^39^
Renal fibrosis	↑^41–45^	unknown
Liver fibrosis	↑^46^	unknown
Pancreatic fibrosis	↑^50^	unknown

Therefore, Dkk-1 appears as an attractive therapeutic target in fibrotic diseases.
